# GlmS and NagB Regulate Amino Sugar Metabolism in Opposing Directions and Affect *Streptococcus mutans* Virulence

**DOI:** 10.1371/journal.pone.0033382

**Published:** 2012-03-16

**Authors:** Miki Kawada-Matsuo, Yusuke Mazda, Yuichi Oogai, Mikihito Kajiya, Toshihisa Kawai, Sakuo Yamada, Shouichi Miyawaki, Takahiko Oho, Hitoshi Komatsuzawa

**Affiliations:** 1 Department of Oral Microbiology, Kagoshima University Graduate School of Medical and Dental Sciences, Kagoshima, Japan; 2 Department of Orthodontics, Kagoshima University Graduate School of Medical and Dental Sciences, Kagoshima, Japan; 3 Department of Preventive Dentistry, Kagoshima University Graduate School of Medical and Dental Sciences, Kagoshima, Japan; 4 Department of Immunology, The Forsyth Institute, Boston, Massachusetts, United States of America; 5 Department of Microbiology, Kawasaki Medical School, Okayama, Japan; 6 Department of Clinical Nutrition, Kawasaki Medical Welfare, Okayama, Japan; Institut Pasteur Paris, France

## Abstract

*Streptococcus mutans* is a cariogenic pathogen that produces an extracellular polysaccharide (glucan) from dietary sugars, which allows it to establish a reproductive niche and secrete acids that degrade tooth enamel. While two enzymes (GlmS and NagB) are known to be key factors affecting the entrance of amino sugars into glycolysis and cell wall synthesis in several other bacteria, their roles in *S. mutans* remain unclear. Therefore, we investigated the roles of GlmS and NagB in *S. mutans* sugar metabolism and determined whether they have an effect on virulence. NagB expression increased in the presence of GlcNAc while GlmS expression decreased, suggesting that the regulation of these enzymes, which functionally oppose one another, is dependent on the concentration of environmental GlcNAc. A *glmS*-inactivated mutant could not grow in the absence of GlcNAc, while *nagB*-inactivated mutant growth was decreased in the presence of GlcNAc. Also, *nagB* inactivation was found to decrease the expression of virulence factors, including cell-surface protein antigen and glucosyltransferase, and to decrease biofilm formation and saliva-induced *S. mutans* aggregation, while *glmS* inactivation had the opposite effects on virulence factor expression and bacterial aggregation. Our results suggest that GlmS and NagB function in sugar metabolism in opposing directions, increasing and decreasing *S. mutans* virulence, respectively.

## Introduction

*Streptococcus mutans* is a commensal bacterium present in the oral cavity and one of the first bacteria to colonize the tooth surface. This bacterium can be isolated from humans with or without dental cavities, suggesting that the oral environment of the host plays an important role in the virulence of *S. mutans*. Growth of this species also changes local environmental conditions, allowing it to attach to the tooth surface by producing an extracellular polysaccharide called glucan, which is involved in the formation of dental plaque [1], [2], [3]. Dental plaque formation is important for the survival and adherence of *S. mutans* to the tooth surface because biofilms allow bacteria to resist immune factors and host-derived antibacterial agents [4]. Sucrose is the most important substrate involved in the synthesis of water-insoluble glucan (mutan), a glucose polysaccharide [5], [6]. *Streptococcus mutans* expresses several glucosyltransferases (GTFs) that produce water-insoluble and/or -soluble glucan molecules (mutan and dextran, respectively) from sucrose. Mutan and dextran function as major matrix components in biofilms [5]. Other sugar metabolic processes are important for maintaining homeostatic bacterial growth and survival. For example, sucrose and other sugars are substrates that drive various metabolic pathways, including glycolysis, peptidoglycan biosynthesis, and teichoic acid biosynthesis [7], [8]. The enzymatic conversion of sugars by *S. mutans*, accompanied by the extracellular production of cell-surface protein antigen (PAc; also known as SpaP), glucan binding protein, dextranase, and acid tolerance factor (H^+^ pump), facilitates acid-catalyzed tooth decay and leads to erosion of the hydroxyapatite of the teeth [5], [6], [9], [10], [11].

Bacteria can uptake and utilize various sugars, including glucose, sucrose, and amino sugars such as glucosamine (GlcN), N-acetylglucosamine (GlcNAc), and N-acetylneuraminic acid. Incorporated sugars are primarily utilized in glycolysis and cell wall biosynthesis [7], [8]. To utilize sugars for such metabolic functions, they are ultimately processed into fructose-6-phosphate (Fru-6P) and glucosamine-6-phosphate (GlcN-6P), which are the initial substrates of glycolysis and peptidoglycan synthesis, respectively [7], [12]. Glutamine-fructose-6-phosphate aminotransferase (GlmS) is involved in the production of GlcN-6P from Fru-6P, and glucosamine-6-phosphate deaminase (NagB) is involved in the production of Fru-6P from GlcN-6P. Specifically, these enzymes possess opposing activities; that is, GlmS catalyzes the conversion of Fru-6P to GlcN-6P, while NagB reverses the catalytic process mediated by GlmS (GlcN-6P to Fru-6P) [7], [13]. The roles of GlmS and NagB have been characterized in several organisms, including *Escherichia coli*, *Bacillus subtilis*, and *Staphylococcus aureus* [7], [8], [12], [13], [14]. However, the mechanism by which these enzymes function in *S. mutans* sugar metabolism is poorly understood. Therefore, in this study we constructed *glmS*- and *nagB*-inactivated mutants to characterize the roles of these two opposing enzymes, which are key factors in glycolysis and cell wall synthesis in *S. mutans*. We also examined the *S. mutans* virulent phenotypes associated with sugar metabolism to link *glmS* and *nagB* to the production of PAc, surface adhesion, and GTF.

## Materials and Methods

### Bacterial strains and growth conditions

The bacterial strains used in this study are listed in Table 1. *Streptococcus mutans* and *E. coli* were grown in trypticase soy broth (TSB) (Becton Dickinson Microbiology Systems, Cockeysville, MD, USA) and Luria-Bertani broth, respectively. Erythromycin (10 µg/mL) and spectinomycin (600 µg/mL) for *S mutans* or ampicillin (100 µg/mL) and erythromycin (300 µg/mL) for *E. coli* were added when necessary. A chemically defined medium (CDM), which was supplemented with glucose (50 mM) as the sole carbon source, was prepared and used in this study (CDM-G50). CDM-G50, which was initially used to culture *S. aureus* [8], [15], consisted of the following five solutions: Solution 1 (20.1 g of Na_2_HPO_4_–12H_2_O; 3 g of KH_2_PO_4;_ 150 mg each of L-aspartic acid, L-glutamic acid, L-isoleucine, L-leucine, L-proline, L-threonine, and L-valine; 100 mg each of L-alanine, L-arginine, glycine, L-histidine, L-lysine, L-methionine, L-phenylalanine, L-serine, L-tryptophan, and L-tyrosine; and 50 mg of L-cysteine dissolved in 700 mL of distilled water and adjusted to pH 7.2); Solution 2 (0.1 mg of biotin, 2 mg of nicotinic acid, 2 mg of D-pantothenic acid, 4 mg of pyridoxal, 4 mg of pyridoxamine dihydrochloride, 2 mg of riboflavin, and 2 mg of thiamine hydrochloride dissolved in 100 mL of distilled water); Solution 3 (20 mg of adenine sulfate and 20 mg of guanine hydrochloride dissolved in 0.1 M hydrochloric acid [HCl] and made up to 50 mL with distilled water); Solution 4 (10 mg of CaCl_2_–6H_2_O, 5 mg of MnSO_4_, and 3 mg of (NH_4_)_ 2_SO_4_-FeSO_4_–6H_2_O dissolved in 10 mL of 0.1 M HCl); and Solution 5 (10 g of glucose and 500 mg of MgSO_4_–7H_2_O dissolved in 100 mL of distilled water). Solutions 1–4 were mixed and the volume was adjusted to 900 mL with distilled water. This mixed solution and Solution 5 were autoclaved separately, then mixed to a total volume of 1 L. When sucrose was used as the sole sugar source, 50 mM sucrose (instead of glucose) was added to Solution 5 to generate CDM-S50. The mixture of Solutions 1–4 (CDM containing no sugars) was used for some bacterial washes.

**Table 1:** Strains and plasmids used in this study.

Strains and plasmids	Relevant characteristics
*Streptococcus mutans*	
UA159	WT laboratory strain
Δ *glmS*	*glmS* (SMU. 1187)^1^ deletion mutant in UA159, Em^r2^
Δ *nagB*	*nagB* (SMU. 636) deletion mutant in UA159, Em^r^
Δ *vicK*	*vicK* (SMU. 1516) deletion mutant in UA159, Spc^r3^
Δ *glmS+vicK*	*glmS* and *vicK* double deletion mutant in UA159, Em^r^, Spc^r^
Δ *nagB+vicK*	*nagB* and *vicK* double deletion mutant in UA159, Em^r^, Spc^r^
Δ *ccpA*	*ccpA*(SMU. 1591) deletion mutant in UA159, Em^r^
*glmS* compl.	*glmS* complementation in MM3011, Em^r^, Spc^r^
*nagB* compl.	*nagB* complementation in MM3007, Em^r^, Spc^r^
*Escherichia coli*	
M15(pREP4)	Host strain for protein expression (Qiagen)
MM1019	pMM1019/*E. coli* M15 for His-tagged GlmS expression, Amp^r4^, Km^r5^
MM1020	pMM1020/*E. coli* M15 for His-tagged NagB expression, Amp^r^, Km^r^
Plasmids	
pQE30	Expression vector for His-tagged protein, Amp^r^ (Qiagen)
pMM1019	*glmS* PCR fragment/pQE30
pMM1020	*nagB* PCR fragment/pQE30
pBluescript SK II (+)	Cloning vector in *E. coli*, Amp^r^
pMM1001	Em^r^ gene harboring the flanking region of *glmS*/pBluescript SK II (+)
pMM1002	Em^r^ gene harboring the flanking region of *nagB*/pBluescript SK II (+)

1 GenBank locus tag obtained from the *S. mutans* genome at the Oral Pathogen Sequence Database site.

2 Erythromycin resistance.

3 Spectinomycin resistance.

4 Ampicillin resistance.

5 Kanamycin resistance.

### Construction of *glmS-*, *nagB-*, *vicK-, and ccpA*-knockout mutants

*Streptococcus mutans* UA159 knockouts were constructed as described previously [16]; the primers used are listed in Table S1. Briefly, the erythromycin resistance (Em^r^) gene derived from *Enterococcus faecalis* was amplified using two specific primers from the plasmid pResEmNot [17] and cloned into pBluescript SK II (+). Next, the target gene flanking regions (*glmS*, *nagB*, or *ccpA*) were amplified with specific primers from the *S. mutans* UA159 genome. The flanking regions were then fused to either end of the Em^r^ gene. Although Em^r^ gene contained no terminator, Em^r^ gene was fused with the downstream fragment including a terminator of each target gene, speculating that Em^r^ gene insertion had no effect on the expression of their downstream genes. After amplification of the Em^r^/flanking gene construct by polymerase chain reaction (PCR), the PCR fragment was transformed into *S. mutans* UA159. The *nagB-* and *ccpA-*knockout mutants were isolated by selection with erythromycin (10 µg/mL), while the *glmS*-knockout mutant was isolated by selection with erythromycin (10 µg/mL) and 10 mM GlcNAc. The *vicK*-knockout mutant was also constructed by the replacement of *vicK* with the spectinomycin resistance gene (Spc^r^) by the method described above. The Spc^r^ gene was amplified from the plasmid pDL55 [18]. The mutation was verified by PCR and immunoblotting. Double knockout mutants (*vicK* combined with *glmS* or *nagB*) were constructed by introducing the *glmS* or *nagB* knockout into the *vicK* mutant using the method described above. 

For genetic complementation, we constructed a DNA fragment to insert the spectinomycin resistance (*spc^r^*) gene and *glmS* or *nagB* into the *ftf* gene coding for fructosyltransferase. First, *spc^r^*, *glmS/nagB*, the N-terminal region of *ftf*, and the C-terminal region of *ftf* were amplified with specific primers. Since amplified-*glmS* and *nagB* contained no their own promoter region, these genes were expressed by using *ftf* promoter. The primers added an extra eight nucleotides to anneal each PCR fragment. The mixture of the N-terminal region of *ftf* and *spc^r^* were then heated at 95°C for 5 min and left to incubate for 30 min at 37°C. DNA polymerase and dNTPs were added to the mixture and allowed to react at 68°C for 15 min; PCR was then performed using both ends of the primers. Finally, all of the fragments were fused by PCR. The product fragment was then transformed into the *glmS* or *nagB* mutant of *S mutans*. By selection for erythromycin and spectinomycin resistance, the complemented strains were isolated. Finally, *spc^r^* and *glmS/nagB* gene insertion into the *ftf* gene was verified by PCR.

### Antiserum production and immunoblotting

Recombinant NagB and GlmS were expressed as 6 × histidine-tagged proteins. The PCR primers are listed in Table S1. DNA fragments encoding the protein, amplified with specific primers for *glmS* and *nagB*, were cloned into pQE30 (Qiagen, Tokyo, Japan) to generate pMM1019 (*glmS*) and pMM1020 (*nagB*). The plasmids were electroporated into *E. coli* M15 (pREP4). The recombinant proteins were purified according to the manufacturer’s instructions. Antisera against the recombinant proteins were obtained by immunizing mice, as described previously [8].

For immunoblotting, exponential phase *S. mutans* cells were collected from a 10-mL culture and washed with PBS. The cells were resuspended in 200 µL of 5% sodium dodecyl sulfate (SDS) and disrupted by ultra-sonication three times at 10-s intervals. The cells were then heated at 100°C for 10 min. After centrifugation, the supernatant was obtained as a whole cell lysate. The concentration of protein in each whole cell lysate was quantified with a BCA protein assay kit (Pierce Biothechnology, Rockford, IL, USA). The same amount of lysate protein was mixed with sample loading buffer, after which 7.5% and 15% SDS-polyacrylamide gel electrophoresis (PAGE) was performed for GlmS and NagB, respectively. Next, the proteins were transferred to a nitrocellulose membrane. The loading of consistent amounts of protein for each sample was confirmed by Coomassie Brilliant Blue staining after an additional SDS-PAGE run. After blocking with 2% skim milk, antiserum for GlmS or NagB was reacted at 37°C for 1 h. After washing the membrane with Tris-buffered saline (20 mM Tris and 137 mM NaCl) and 0.05% Tween 20, horseradish peroxidase-conjugated anti-mouse IgG was reacted at 37°C for 1 h. The membrane was washed five times, and the protein band that reacted with the antiserum was detected using a chemiluminescence detection system (PerkinElmer, Waltham, MA, USA). 

### Growth kinetics of wild-type (WT) and mutant *S. mutans*

Overnight cultures of WT and mutant *S. mutans* were harvested by centrifugation at 8000 × *g* for 5 min, and the cells were resuspended in equivalent volumes of TSB, BHI, or CDM-G50 to adjust the OD_660_ to 1.0. Next, the suspension was diluted with the appropriate medium to 10^6^ cells/mL (1000-fold dilution). Growth was monitored using a SPECTRA max 340PC^384^ (Molecular Devices, Sunnyvale, CA, USA) with a 96-well microtiter plate. When required, various concentrations of GlcNAc were added to the medium. The doubling time was calculated based on the formulas ln Z – ln Z_0_ = *k* (*t* – *t_0_*), where *k* is the growth rate, and *g* = 0.693/*k*×60, where *g* is the doubling time (min), as described previously [16].

### Quantitative PCR analysis of gene expression

A small aliquot of *S. mutans* cultured overnight was inoculated into fresh medium and grown at 37°C; bacterial cells at various stages of growth were collected. Total RNA was extracted from the cells using a FastRNA Pro Blue kit (MP Biomedicals, Solon, OH, USA), according to the manufacturer’s protocol. One microgram of total RNA was reverse-transcribed to cDNA using a first-strand cDNA synthesis kit (Roche Diagnostics, Tokyo, Japan). Using the cDNA as template, quantitative PCR was performed using the MyiQ2 system (Bio-Rad Laboratories, Tokyo, Japan). Primers for *gyrA*, *glmS*, *nagB*, *gtfB*, *gtfC*, *spaP* (encoding PAc), *ccpA*, and *fruA* were synthesized and used to determine the optimal expression conditions. Primers for the two-component systems (TCSs) were also synthesized. *gyrA* was used as an internal control. All primers used in this study are shown in Table S1. For quantitative PCR, three independent experiments were performed, and the mean ± SD was calculated.

### Northern blot analysis

Total RNA from WT and mutant UA159 cells grown in CDM-G50 with or without GlcNAc was extracted as described above, and 10 µg of each sample were used for agarose gel electrophoresis. Electrophoresis and transfer to a nylon membrane were performed as described previously [19]. Hybridization and DIG labeling were performed according to the manufacturer’s protocol (Roche Diagnostics). DIG-labeled PCR fragments of *glmS* or *nagB* were used as probes for hybridization. After pre-hybridization at 42°C for 30 min, hybridization was performed at 42°C for 16 h. The membrane was then washed with 5 X SSC and 0.5% SDS (twice for 5 min each at room temperature) and then with 0.2 X SSC and 0.5% SDS (twice for 15 min each at 42°C). The reacted bands were visualized by the addition of substrate, according to the manufacturer’s protocol.

### Expression of *S. mutans* virulence factors

Virulence factor expression was investigated by quantitative PCR and immunoblotting. Exponential phase *S. mutans* cells (OD_660_ = 0.5) were harvested. For quantitative PCR, total RNA was extracted and cDNA was synthesized as described above. The primers used for *gtfB*, *gtfC*, and *spaP* are listed in Table S1. For immunoblotting, the samples were prepared as described above. Antisera against *S. mutans* GTF-I and PAc were obtained previously [20]. Because of the strong similarity between GTF-I (*gtfB*) and GTF-SI (*gtfC*) (73% amino acid identity), the antiserum against GTF-I also recognized GTF-SI.

### Evaluation of biofilm formation

Overnight cultures were diluted 1∶10 in fresh TSB and grown to the exponential phase (OD_660_ = 0.35). Next, the cells were harvested by centrifugation and resuspended in CDM. Aliquots (20 µL) of exponential stage cells were inoculated into wells containing 200 µL of fresh CDM-G50 with 50 mM sucrose or CDM-S50 in the presence or absence of 1 mM GlcNAc. The plates were incubated at 37°C with 5% CO_2_ for 16 h. To monitor bacterial growth, the OD_660_ was measured prior to safranin staining. The medium was then removed, and the wells were washed three times with distilled water. Finally, the biofilm cells were stained with 0.1% safranin for 10 min [21]. After three additional washes with distilled water, biofilm quantification was performed by evaluating the absorbance of each well at 490 nm. All experiments were performed in triplicate.

### Electron microscopy

For scanning electron microscopy, WT and mutant UA159 cells were grown on a glass disk in TSB with 2% sucrose in the presence or absence of GlcNAc. After incubating overnight, the biofilm cells were washed twice with PBS. Next, a bacterial cell suspension was mounted on a glass coverslip and fixed with 2.5% glutaraldehyde and 1% osmium tetroxide (OsO_4_). After dehydration using a graded ethanol series, drying using the critical-point procedure, and coating with gold-palladium, each specimen was examined under a JEOL JSM-6340F scanning electron microscope at 10 kV.

### Saliva-induced aggregation assay

For the saliva-induced aggregation assay, exponential phase *S.* *mutans* cells grown in TSB with or without GlcNAc were suspended in aggregation buffer at an OD_660_ of approximately 1.0. Unstimulated whole saliva was collected from a single donor (male, 47 years of age) in an ice-chilled plastic tube and subjected to centrifugation at 12000×*g* for 15 min. Either whole saliva (100 µL) or 10 µL of salivary agglutinin (0.5 mg/mL) was mixed with 1 mL of the cell suspension. Salivary agglutinin was purified as described previously [22]. CaCl_2_ was also added to the salivary agglutinin mixture to a final concentration of 1 mM. Bacterial aggregation was determined by monitoring the change in OD_550_ at 37°C with a spectrophotometer. All experiments were performed in triplicate.

## Results

### Construction of *glmS-* and *nagB-*knockout mutants

We constructed *S. mutans glmS*- and *nagB*-knockout mutants using recombination to replace *glmS* or *nagB* with the Em^r^ gene (Table 1). The knockouts and their complementation were verified by PCR and immunoblotting using GlmS- or NagB-specific antibodies (Fig. S1).

**Figure 1:**
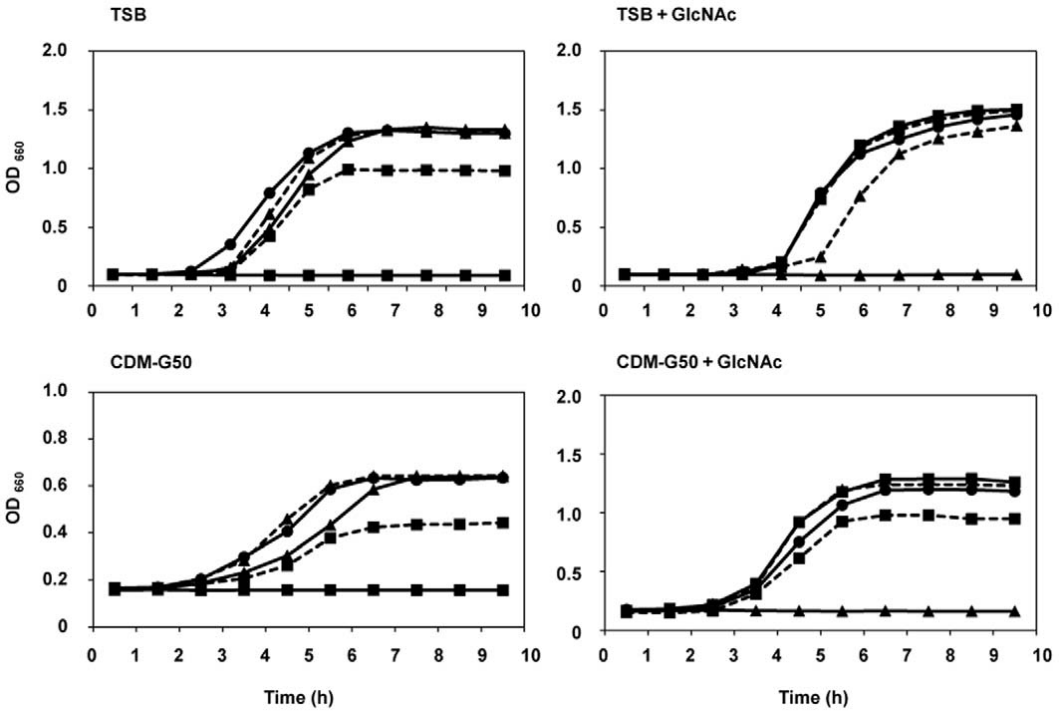
*Streptococcus mutans* growth curves in various bacterial media. A small aliquot of an overnight culture of WT (circle), *glmS* mutant (square), *nagB* mutant (triangle), *glmS* complement strain (square, dashed line) or *nagB* complement strain (triangle, dashed line) cells was inoculated into TSB or CDM-G50 with or without 10 mM GlcNAc and incubated at 37°C with 5% CO_2_. Growth was monitored by measuring the OD_660_.

Since GlcNAc significantly affected the growth of the *glmS*-knockout mutants in *S. aureus* [8], we investigated the growth of these *S. mutans* mutants in the presence or absence of 10 mM GlcNAc (Fig. 1). The doubling time and final OD of the WT and mutant cells grown under various conditions are shown in Table 2. In the absence of GlcNAc in TSB or CDM-G50, the *glmS* mutant did not grow, but in the presence of 10 mM GlcNAc, the *glmS* mutant replicated at a rate equal to that of WT. The *glmS*-knockout mutant strain complemented with *glmS* grew in the absence of GlcNAc, although its doubling time (60.4 min in TSB and 137.6 min in CDM-G50) was higher than that (45.9 min in TSB and 112.3 min in CDM-G50) of the WT strain. In contrast, the presence of 10 mM GlcNAc inhibited *nagB* mutant growth. The *nagB*-knockout mutant strain complemented with *nagB* grew in the presence of GlcNAc. On the other hand, in the absence of GlcNAc, the uncomplemented *nagB* mutant grew, but its doubling time (51.8 min in TSB and 132.7 min in CDM-G50) was higher than that of wild type (45.9 min in TSB and 112.3 min in CDM-G50).

**Table 2:** Doubling times of UA159, *glmS* or *nagB* deletion mutant and its complement strains grown in TSB or CDM-G50.

	TSB		TSB+GlcNAc		CDM-G50		CDM-G50+GlcNAc	
	DT 1	final OD	DT	final OD	DT	Final OD	DT	final OD
UA159	45.9±1.0	1.30±0.14	39.8±1.3	1.46±0.02	112.3±6.1	0.63 ±0.01	74.2±5.9	1.18±0.02
Δ *glmS*	ND	0.09±0.01	37.5±1.6	1.50±0.02	ND	0.16±0.01	76.4±6.4	1.26±0.04
Δ *nagB*	51.8±0.9	1.33±0.01	ND	0.10±0.01	132.7±16.1	0.64±0.06	ND	0.16±0.03
Δ *glmS*::*glmS*	60.4±2.3	0.98±0.01	41.8±1.0	1.50±0.01	137.6±1.4	0.44±0.01	75.8±5.9	0.95±0.05
Δ *nagB*::*nagB*	48.2±2.2	1.30±0.02	56.9±0.1	1.36±0.03	115.3±5.8	0.64±0.01	71.9±4.4	1.23±0.02

1 Doubling time (DT) was calculated based on the formulas ln Z – ln Z_0_ = *k* (*t* – *t_0_*), where *k* is the growth rate, and *g* = 0.693/*k*×60, where *g* is the doubling time (min). Values are the mean ± standard deviation obtained from three independent experiments.

### Effect of GlcNAc on GlmS and NagB expression

**Figure 2:**
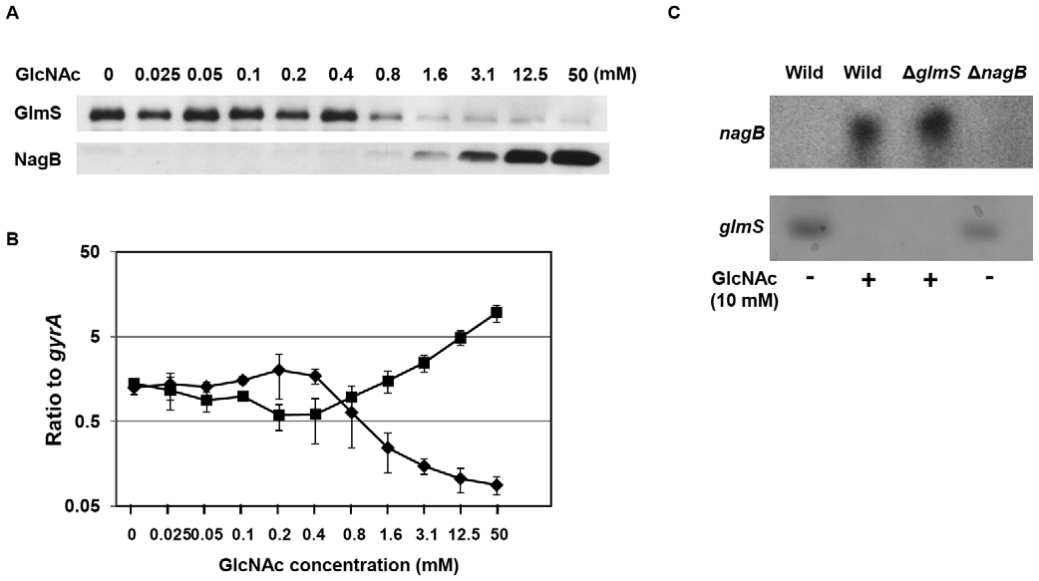
Effect of GlcNAc on GlmS and NagB expression. After washing a WT UA159 overnight culture, a small aliquot was inoculated into CDM-G50 containing various concentrations of GlcNAc and then incubated at 37°C with 5% CO_2_. When the sample reached an OD_660_ of 0.5, the cells were collected. Next, the samples were prepared for immunoblotting (A), quantitative PCR (B), and Northern blotting (C) as described in the Materials and Methods. Panel B: The diamonds and squares represent *glmS* and *nagB* expression, respectively.

We investigated the expression of NagB and GlmS in the presence of various concentrations of GlcNAc in CDM-G50 (Fig. 2). Since TSB contains several sugars, we used CDM-50 for this assay to control the glucose/GlcNAc ratio. Exponential growth phase *S. mutans* cells (OD = 0.8) in CDM-G50 containing various concentrations of GlcNAc were prepared. In the absence of GlcNAc, NagB was not detected by immunoblotting, while GlmS was expressed. GlmS expression was not altered by GlcNAc at concentrations below 0.4 mM. However, at a GlcNAc concentration of 0.8 mM or higher, GlmS expression decreased gradually until the GlcNAc concentration reached 12.5 mM, at which point GlmS expression was no longer detected. In contrast, while NagB was not expressed at GlcNAc concentrations of less than 0.8 mM, its expression at a GlcNAc concentration of 1.6 mM or higher was detected, suggesting that protein expression increased gradually under increasing GlcNAc concentrations. Quantitative PCR and immunoblotting corroborated these results, demonstrating that the expression of both GlmS and NagB was altered in the presence of 1.6 mM GlcNAc (Fig. 2B). Northern blotting confirmed these results, showing that significant amounts of *glmS* transcript were present in CDM-G50 in the absence of GlcNAc, but that the addition of GlcNAc reduced the transcript level (Fig. 2C). In contrast, *nagB* transcript was detected in CDM-G50 in the presence of GlcNAc, but was abolished in the absence of GlcNAc (Fig. 2C).

**Figure 3:**
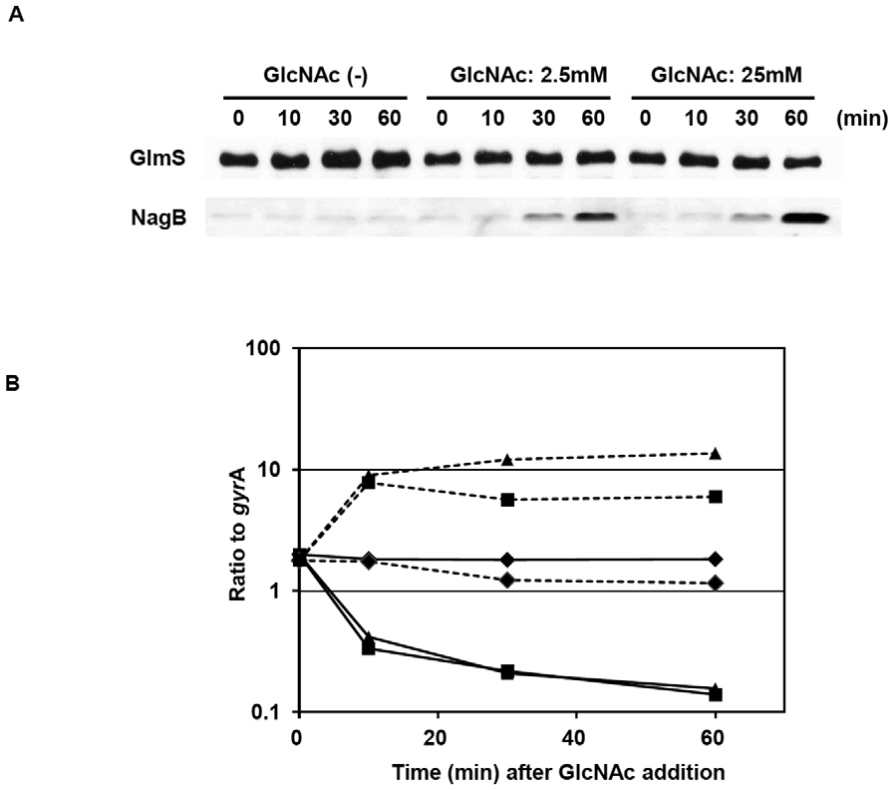
Time course of GlmS and NagB expression after the addition of GlcNAc. *Streptococcus mutans* UA159 was grown in CDM-G50 at 37°C with 5% CO_2_. When the cells reached an OD_660_ of 0.5, GlcNAc was added at 2.5 or 25 mM. Cells were collected 10, 30, and 60 min after the addition of GlcNAc. Next, the cells were prepared for immunoblotting (A) and quantitative PCR (B) as described in the Materials and Methods. Panel B: The symbols represent samples without GlcNAc (diamonds), with 3 mM GlcNAc (squares), and with 25 mM GlcNAc (triangles). Normal and dashed lines represent *glmS* and *nagB* expression, respectively.

We next investigated NagB and GlmS expression over time after the addition of GlcNAc. Ten minutes after GlcNAc addition, the *glmS* transcript level was significantly decreased, while the *nagB* transcript level was increased (Fig. 3B). By immunoblotting, NagB was detected after 30 min, while the amount of GlmS did not change up to 60 min after the addition of 25 mM GlcNAc (Fig. 3A). These differential responses can be attributed to the rate of degradation of the protein and mRNA.

### Effects of *glmS* and *nagB* on virulence factor expression

**Figure 4:**
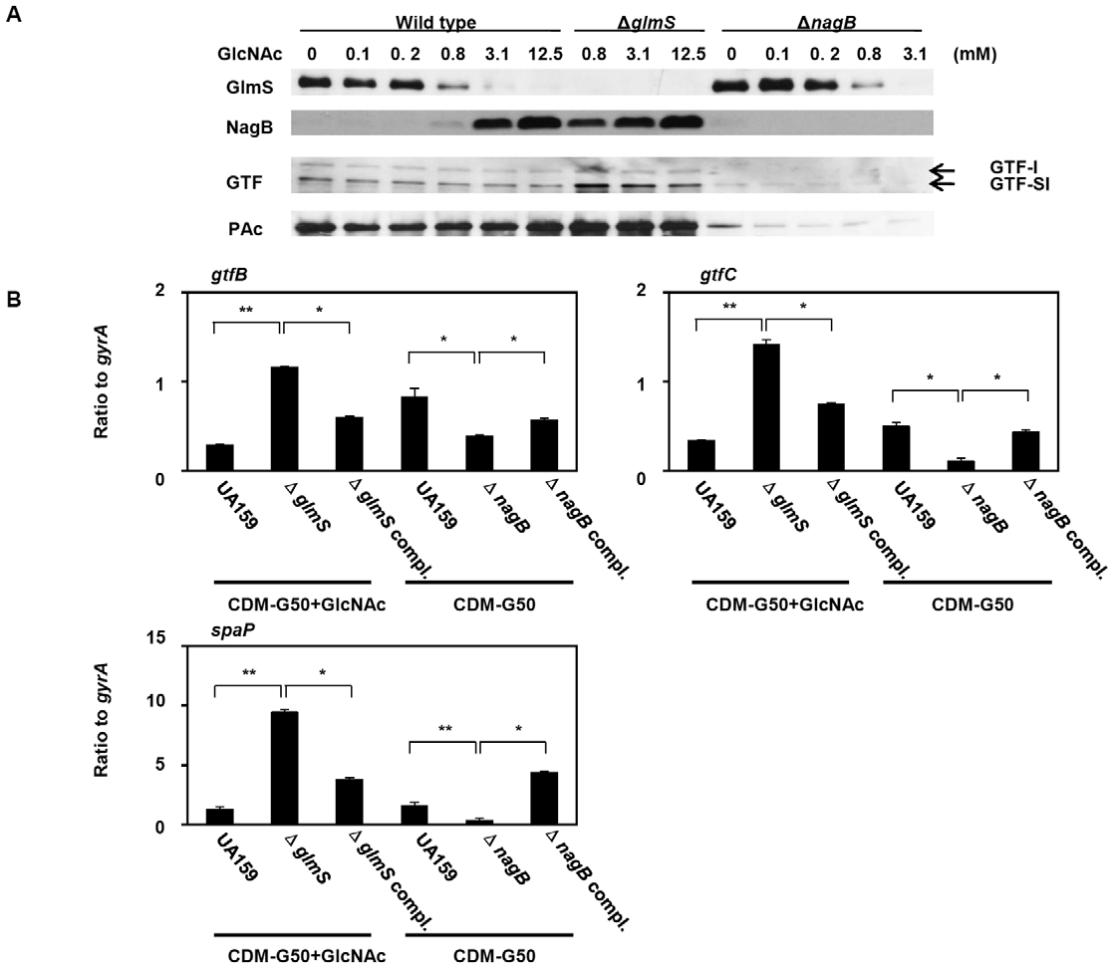
GlmS, NagB, and virulence factor expression in WT UA159 cells, its deletion mutants, and complementation strains. After washing overnight cultures of WT and mutant cells, a small aliquot of each was inoculated into CDM-G50 containing various concentrations of GlcNAc and incubated at 37°C with 5% CO_2_. At an OD_660_ of 0.5, the cells were collected and prepared for immunoblotting (A) and quantitative PCR (B) as described in the Materials and Methods. *p < 0.05, as determined by Tukey’s HSD; **p < 0.005, as determined by Tukey’s HSD.

Since numerous studies have explored the effects of sugars on virulence factors, including glucan synthesis, biofilm formation, and acid production, in *S. mutans* [5], [6], [23], we investigated the expression of virulence factors in our *glmS* and *nagB* mutants in the exponential growth phase (OD = 0.8) in CDM-G50 with or without 10 mM GlcNAc. GlmS or NagB expression was completely abolished in the *glmS* and *nagB* mutants, respectively. However, GlmS expression in the *nagB* mutant as well as NagB expression in the *glmS* mutant was similar to that in WT bacteria, indicating that expression of the enzyme targeted for knockout was only abolished by its particular mutation (and not by the mutation of other enzymes) (Fig. 4A). Expression of the virulence proteins GTF-I (involved in water-insoluble glucan synthesis), GTF-IS (involved in water-soluble and -insoluble glucan synthesis), and PAc (a cell-surface antigen that mediates adhesion to hydroxyapatite and salivary components) in mutants grown in CDM-G50 with or without GlcNAc was analyzed by immunoblotting and quantitative PCR (Fig. 4A and B). We also investigated the expression of GlmS and NagB early in the exponential growth phase (OD = 0.4) and in the stationary phase (OD = 1.0) and obtained results similar to those obtained in the mid-exponential phase (OD = 0.8) (data not shown). Immunoblotting revealed that the PAc and GTF concentrations were increased in the *glmS* mutant and decreased in the *nagB* mutant compared to wild type. In the WT strain, GTFs and PAc expression was slightly decreased by GlcNAc (Fig. 4A). In quantitative analysis, the expression of the *gtfB* (GTF-I), *gtfC* (GTF-IS), and *spaP* (PAc) in the WT strain were decreased at 2.8-, 1.5- and 1.2-fold, respectively, by addition of GlcNAc. In the *nagB* mutant, the expression of the *gtfB* (2.2-fold lower), *gtfC* (4.8-fold lower), and *spaP* (4.2-fold lower) were decreased compared to the WT strain. In the *glmS* mutant, the expression of the *gtfB* (4.0-fold higher), *gtfC* (4.2-fold higher), and *spaP* (7.5-fold higher) were increased compared to the WT strain. In the *glmS*-complemented strain, the expression of these genes was reduced compared to the *glmS* mutant, but not fully recovered to those of the WT strain. Also, in the *nagB*-complemented strain, the expression of these genes was increased compared to the *nagB* mutant, but the expression of *gtfB* and *gtfC* was not fully restored (Fig. 4B).

### Association between TCS and virulence factor expression in the *glmS* and *nagB* mutants

**Figure 5:**
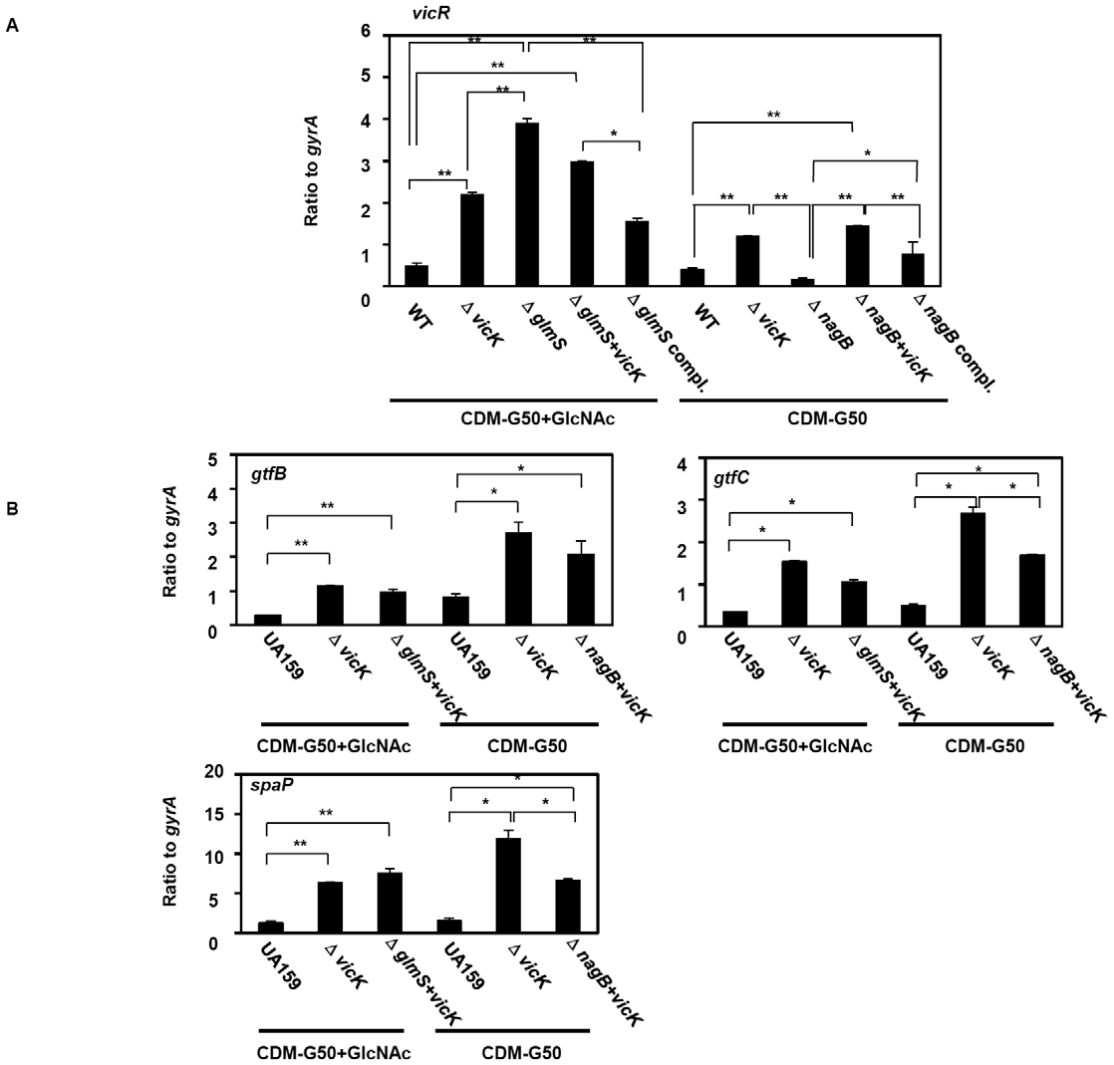
Association of VicRK with virulence factor expression in the *glmS* and *nagB* mutants. After washing overnight cultures of WT and mutant cells, a small aliquot of each was inoculated into TSB containing 50 mM GlcNAc and incubated at 37°C with 5% CO_2_. When the samples reached an OD_660_ of 0.5, cells were collected and prepared for quantitative PCR as described in the Materials and Methods. (A) *vicR* expression in WT UA159, *vicK* mutant, *glmS* deletion mutant, *nagB* deletion mutant, *glmS+vicK* double mutant, *nagB+vicK* double mutant, *glmS* complement strain, and *nagB* complement strain cells. *p < 0.05, as determined by Tukey’s HSD; **p < 0.005, as determined by Tukey’s HSD. (B) *gtfB*, *gtfC*, and *spaP* expression in UA159, *vicK* mutant, *glmS+vicK* double mutant, and *nagB+vicK* double mutant strains. *p < 0.05, as determined by Tukey’s HSD; **p < 0.005, as determined by Tukey’s HSD.

In *S. mutans* UA159, 15 sets of TCSs (including one orphan TCS) were identified in the genome, some of which are known to be associated with virulence factor expression, including *gtfs* [16], [24]–[27]. In this study, the *glmS* and *nagB* mutants showed altered *spaP* and *gtf* expression. Therefore, we investigated whether this altered expression was due to the effect of the inactivation of *glmS* and *nagB* on TCS expression. First, we investigated the expression of all TCSs in the *glmS* and *nagB* mutants (Fig. S2) and found that three TCSs (*vicR, comE* and SMU.1815) expression was increased in the *glmS* mutant, while only *vicR* was decreased in the *nagB* mutant, showing only *vicR* expression was changed in *glmS* and *nagB* mutant. The expression of *vicR* in the *glmS* and *nagB* mutants was restored in the respective complemented strains (Fig. 5A). *vicRK* was previously shown to affect *gtfB* and *gtfC* expression [25], [26]. Next, we constructed a *vicK* mutant to investigate the interaction of *vicRK* with *spaP*, *gtfB*, and *gtfC* expression. Since *vicR* is known to be an essential gene [16], we constructed a *vicK* mutant and found that the absence of *vicK* increased *vicR* expression (Fig. 5A). The *vicK* mutation in the *nagB* mutant as well as in the wild type showed the increase of *vicR* expression. In the case of *vicK* mutation in the *glmS* mutant, *vicR* expression is similar to that of the *glmS* mutant, although its expression was increased compared to wild type. In addition, *spaP*, *gtfB*, and *gtfC* expression was increased in the *vicK* mutant (Fig. 5B). Therefore, *vicRK*, whose expression was increased in the *glmS* mutant and decreased in the *nagB* mutant, appeared to modulate the virulence genes *spaP*, *gtfB*, and *gtfC.* Furthermore, we investigated the expression of virulence factors in the double mutants (*vicK* combined with *glmS* or *nagB*). In the *vicK* and *nagB* double mutant, *spaP*, *gtfB*, and *gtfC* expression was increased compared to that in the *nagB* mutant, although *nagB* mutation in the *vicK* mutant reduced the expression of these factors compared to that of the *vicK* mutant (Fig. 4B and Fig. 5B). The *vicK* and *glmS* double mutant showed similar expression to that in the *vicK* or *glmS* mutant (Fig. 4B and Fig. 5B).

### Effect of CcpA on the expression of *glmS* and *nagB*

**Figure 6:**
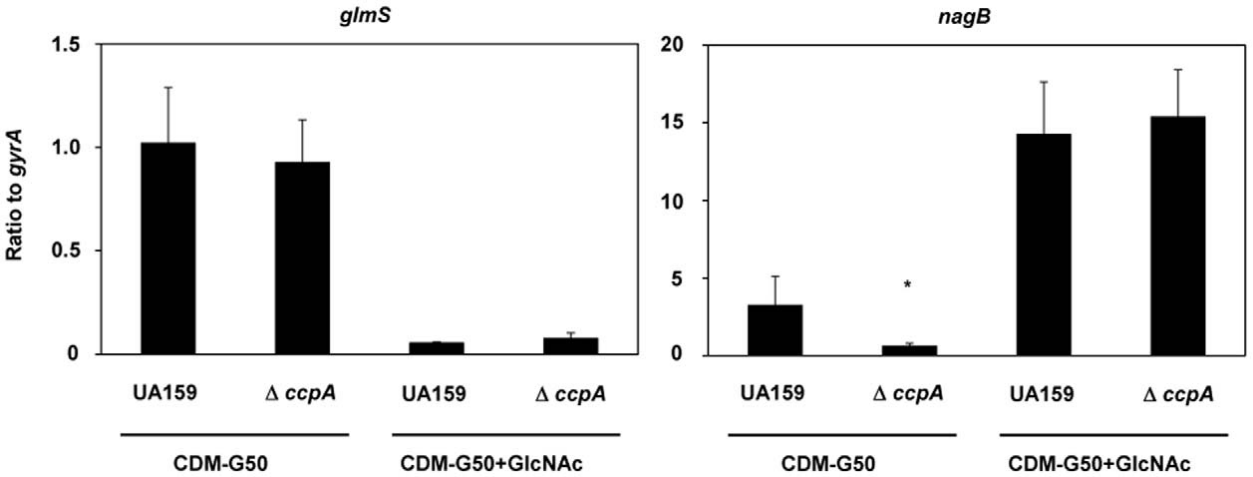
*glmS* and *nagB* expression in *ccpA* mutant cells. After washing overnight cultures of WT and *ccpA* mutant cells, a small aliquot of each was inoculated into CDM-G50 with or without 50 mM GlcNAc and incubated at 37°C with 5% CO_2_. When the samples reached an OD_660_ of 0.5, cells were collected and prepared for quantitative PCR as described in the Materials and Methods. *p < 0.05, compared to WT UA159 as determined by a *t*-test.

Since CcpA plays a central role in carbon catabolite repression and affects the virulence of *S. mutans* [28], we investigated whether CcpA affects the expression of *glmS* and *nagB*. In *ccpA* mutant cells grown in CDM-G50 with or without 10 mM GlcNAc, *glmS* expression was unchanged compared with that in wild type (Fig. 6). The expression of *nagB* in the *ccpA* mutant grown in CDM-G50 was slightly decreased compared with that in wild type; however, its expression increased upon the addition of GlcNAc to a similar level as in wild type. *gtfBC* and *spaP* expression was unchanged in the *ccpA* mutant (data not shown).

### Biofilm formation in the *nagB* and *glmS* mutants

**Figure 7:**
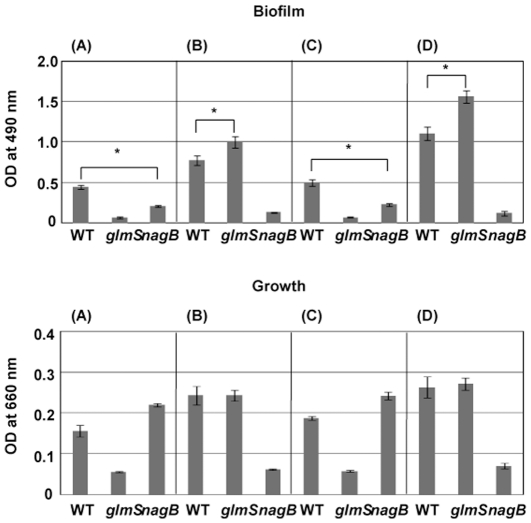
Biofilm formation by WT UA159, *glmS* mutant, and *nagB* mutant cells. After washing overnight cultures of WT and mutant cells, a small aliquot of each was inoculated into each of four solutions: (A) CDM-G50 with 50 mM sucrose, (B) CDM-G50 with 50 mM sucrose and 10 mM GlcNAc, (C) CDM containing 50 mM sucrose (no glucose), and (D) CDM containing 50 mM sucrose and 10 mM GlcNAc (no glucose). The cultures were then incubated at 37°C with 5% CO_2_. After 24 h, the OD_660_ was measured (lower panel). After washing the wells with PBS several times, the cells were stained with 0.1% safranin. The OD_490_ was measured to quantify biofilm formation (upper panel). All experiments were performed in triplicate. *p < 0.05, compared to WT UA159 as determined by a *t*-test.

Since the *glmS* and *nagB* mutants showed altered expression of *gtfs*, which plays a pivotal role in bacterial adhesion to the tooth surface via insoluble glucan formation from sucrose, we investigated biofilm formation in these mutants using CDM containing either sucrose (CDM-S50) or glucose and sucrose (CDM-G50 with 50 mM sucrose) (Fig. 7). In both types of media, biofilm formation in the *nagB* mutant was reduced compared to that in wild type. In CDM containing sucrose or glucose and sucrose plus 10 mM GlcNAc, biofilm formation by the *glmS* mutant exceeded that of wild type. We also evaluated biofilm formation in TSB and BHI media with or without 10 mM GlcNAc and found patterns similar to those in the CDM-based medium (data not shown).

Next, we investigated the expression of GlmS, NagB, GTFs, and PAc in biofilm cells by immunoblotting and quantitative PCR (Fig. S3). Although the expression level of these factors were different between planktonic and biofilm condition, the expression pattern was almost similar between them. GlmS expression in wild type and the *nagB* mutant decreased in the presence of GlcNAc, while NagB expression in wild type and the *glmS* mutant was increased in the presence of GlcNAc. The expression of *gtfB*, *gtfC* and *spaP* were decreased in the *nagB* mutant, showing a similar pattern with that of planktonic condition. However, in the *glmS* mutant, *gtfB* expression in biofilm cells was decreased compared to that of the WT strain, while its expression in planktonic cells was increased. The expression of *gtfC* and *spaP* in the *glmS* mutant under biofilm was increased and showed a similar pattern to that of planktonic condition.

**Figure 8:**
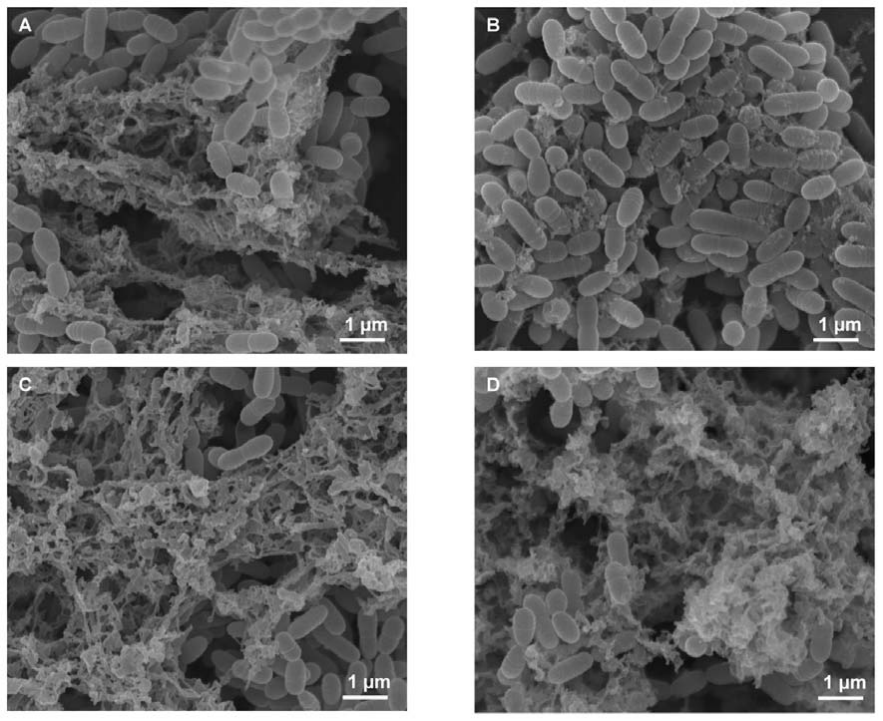
Scanning electron microscopic observation of the mutant biofilm cells. *Streptococcus mutans* WT (A and C), *nagB* mutant (B), and *glmS* mutant (D) cells were grown on a glass disk in TSB with 2% sucrose in the presence (C and D) or absence (A and B) of GlcNAc. After incubation overnight, the biofilm cells were washed twice with PBS. The cells were then fixed with 2.5% glutaraldehyde and 1% OsO_4_.

Electron microscopic observation revealed that the amount of extracellular matrix in the *nagB* mutant was reduced, while the *glmS* mutant had large amounts of extracellular matrix compared with wild type (Fig. 8).

### Saliva-induced aggregation of the *nagB* and *glmS* mutants

**Figure 9:**
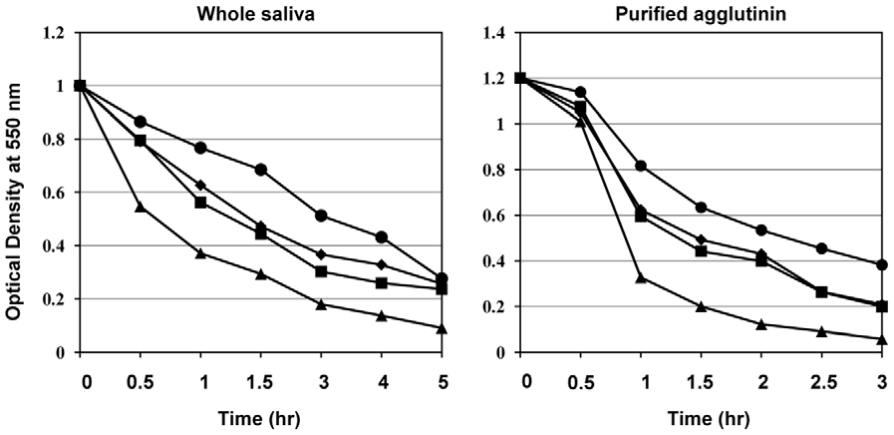
Saliva-induced aggregation of UA159 and the mutants. Exponential phase WT cells grown in CDM-G50 (square) or CDM-G50 containing 10 mM GlcNAc (diamond), *glmS* mutant cells grown in CDM-G50 containing 10 mM GlcNAc (triangle), and *nagB* mutant cells grown in CDM-G50 (circle) were suspended in aggregation buffer at an OD_660_ of 1.0. Whole saliva or purified salivary agglutinin was added to the bacterial suspension, and the OD_550_ was monitored.

Since PAc is involved in saliva-induced aggregation in *S. mutans* [29], we performed a saliva-induced aggregation assay using whole saliva and purified salivary agglutinin with wild type and the *nagB* and *glmS* mutants (Fig. 9). Under both conditions, the *glmS* mutant showed strong aggregation while the *nagB* mutant showed weak aggregation when compared to wild type. There was no difference in salivary aggregation between the WT strain grown in TSB with or without GlcNAc. We also performed an aggregation assay using whole saliva from three other volunteers and found the same results (data not shown).

## Discussion

**Figure 10:**
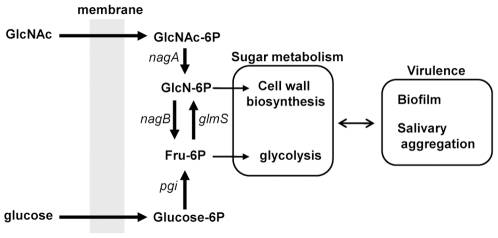
Proposed sugar distribution mediated by GlmS and NagB. Glucose and/or GlcNAc was incorporated into the cytoplasm by PTS and then processed for cell wall synthesis and glycolysis. GlcNAc, N-acetylglucosamine; GlcN, glucosamine; IPS, intracellular polysaccharide; GTF, glucosyltransferase.

In this study, we found that GlmS and NagB coordinately regulated the conversion between glucose and GlcNAc and that failure of this regulation affected the expression of virulence factors in *S. mutans*. Since significant growth inhibition was observed in the *glmS* mutant grown in the absence of GlcNAc and in the *nagB* mutant grown in the presence of GlcNAc (Fig. 1), each enzyme is considered essentially under specific conditions (with or without GlcNAc) in *S. mutans*. In some bacterial species, GlmS and NagB function to distribute sugar substrates to various metabolic pathways, including glycolysis and peptidoglycan biosynthesis [7], [8]. Based on the results of the present study and supported by previous reports [7], [8], we propose a similar mechanism for sugar distribution involving two factors in *S. mutans* (Fig. 10). GlcN-6P is mainly utilized for peptidoglycan biosynthesis, but a high concentration of GlcN-6P in the bacterial cytoplasm is toxic [30], [31]. Therefore, the controlled conversion of GlcN-6P to a non-toxic molecule (Fru-6P) is required, resulting in increased NagB expression. Additionally, the production of GlcN-6P from Fru-6P is suppressed by reducing GlmS expression. In the absence of GlcNAc, GlcN-6P is solely synthesized from Fru-6P (mediated by GlmS), resulting in high GlmS and low NagB expression. Thus, in the *glmS* mutant, the supply of GlcN-6P converted from Fru-6P was abolished, forcing the bacteria to rely on GlcNAc to survive and grow. The *nagB* mutant, in the presence of high GlcNAc concentrations, was unable to process sufficient levels of toxic GlcN-6P, causing growth inhibition. This suggests that tight regulation of GlmS and NagB is critical for sugar metabolism in *S. mutans*. Previously, we demonstrated that *nagB*-knockout *S. aureus* did not show strong growth inhibition in the presence of GlcNAc [8]. This difference is likely due to differences in GlmM activity, which mediates the conversion of Glc-6P to GlcN-1P, the first substrate in peptidoglycan synthesis. One possibility is that increased GlmM activity in *S. aureus* reduces the amount of GlcN-6P. Further study will be required to clarify the difference.

The precise mechanism of GlmS and NagB regulation in *S. mutans* remains unclear. In *B. subtilis*, *glmS* regulation involves the self-degradation of *glmS* mRNA [32], [33], [34], [35]. This regulation, known as ribozyme regulation, involves self-cleavage induced by excess GlcN-6P, a product of the GlmS reaction. A core region consensus sequence 200–300 bp upstream of the *glmS* coding region is required for ribozyme activity. This region of the mRNA can bind to GlcN-6P, inducing self-cleavage and inhibiting the translation of *glmS*. There is no consensus sequence (homologous to the *B. subtilis* core region of ribozyme) upstream of the *glmS* coding region in *S. mutans*. Also, the transcriptional start site in *glmS*, identified by rapid amplification of cDNA ends (RACE) experiments, is 87 bp upstream of the coding region (Fig. S4). This suggests that *S. mutans glmS* has no ribozyme activity. In *Enterobacteriaceae*, *E. coli*, *Salmonella typhimurium*, and *Yersinia pseudotuberculosis*, small RNAs (*glmY* and *glmZ*) were found to regulate *glmS* expression [36], [37]. The small RNA *glmZ* binds to *glmS* mRNA and inhibits translation of the *glmS* gene [36], [37]. *glmY* is also a small RNA that regulates *glmZ* expression by binding to *glmZ* mRNA directly [36], [37]. Although the small RNAs *glmZ* and *glmY* can down-regulate the translation of *glmS* in *Enterobacteriaceae*, it is unknown whether the same system is present in *S. mutans*. In addition, little is known regarding whether the regulation of NagB involves self-degradation or small RNAs in *S. mutans*; thus, additional studies are required. Furthermore, we searched for possible regulatory protein binding sites in the promoter regions of both genes and found none.

In this study, we investigated several phenotypes using *nagB* and *glmS* mutants. Of the phenotypes observed, the most striking was that the *nagB* mutant produced a reduced biofilm, while the *glmS* mutant had slightly elevated levels, compared with wild type (Fig. 7). This was caused by decreased amounts of GTFs (GTF-I and -SI) and PAc in the *nagB* mutant and increased amounts in the *glmS* mutant. We confirmed that these changes were constant during growth (data not shown). We also investigated the effect of pH on the expression of these genes and found similar pH values of the medium in which the mutants grew compared with wild type (data not shown). These results indicate that *glmS* or *nagB* inactivation altered the expression of these virulence factors. In *S. mutans*, three GTFs have been identified that are known to be involved in sucrose-dependent biofilm formation [5]. Besides these factors, *gbpB* and *atlA* were also reported to be associated with biofilm formation [21], [25], [38], although the major factors for sucrose-dependent biofilm formation are GTF-I and -SI, which synthesize water-insoluble glucan. It was previously reported that *gbpB* was regulated by VicR and associated with the initiation of biofilm formation [38]. We investigated the expression of these two factors and found that *gbpB* expression in the *glmS* and *nagB* mutants was similar to that of *gtfBC*, while *atlA* expression was unchanged in both mutants (data not shown). In addition, we found that both mutants had altered saliva-induced aggregation (Fig. 9). PAc is responsible for surface hydrophobicity and sucrose-independent adherence to tooth surfaces and salivary aggregation [11], [29], [39], [40]. Salivary agglutinin, gp340, binds to PAc in *S. mutans*, resulting in aggregation [22], [40]. Therefore, altered salivary aggregation activity may be associated with PAc expression in the mutants. One orphan response regulator (*gcrR*) and one TCS (*vicRK*) were shown to alter *gtfB* and *gtfC* expression in *S. mutans* [24], [25], [26]. We investigated the expression of these regulators in WT and mutant strains, and found that *vicRK* expression was altered in the *glmS* and *nagB* mutants, while *gcrR* expression was unchanged (data not shown). We also found that the *vicK* mutants had increased expression of *vicR*, resulting in increased expression of *spaP*, *gtfB*, and *gtfC*. The mechanism underlying the increased expression of *vicR* in the *vicK* mutant is not well understood. A similar result was found in which the knockout of *ciaH* increased *ciaR* expression [27]. Furthermore, in the *glmS+vicK* double mutant, the expression of *gtf* and *pac* was comparable to that in the *vicK* and *glmS* single mutants, while their expression in the *nagB+vicK* double mutant was increased compared to that in the *nagB* mutant. However, *nagB* mutation in the *vicK* mutant reduced *gtf* and *pac* expressions, this implies that other factor, which is dependent for *nagB*, but independent for *vicR*, is involved in the expression of virulence factors. It was previously shown that the consensus region of the VicR binding site (TGTWAHNNNNNTGTWAH) is upstream of *gtfB* and *gtfC* [25]. We also found the consensus region upstream (136 bp) of the *spaP* transcriptional start site (data not shown). This suggests that the altered expression of *gtfB*, *gtfC*, and *spaP* in the *glmS* and *nagB* mutants is caused by VicRK, although the mechanism underlying the altered expression of *vicRK* in these mutants has not been determined.

Recently, it was shown that CcpA, a transcriptional regulator, affects virulence [28]. CcpA plays a central role in carbon catabolite repression, together with the HPr and PTS system. HPr is activated by HPr kinase, which is activated by an enhanced level of glycolytic intermediates, including fructose-1,6-bisphosphate or glucose-6P. Next, CcpA and HPr form a complex, resulting in enhanced binding to catabolite responsive elements in the promoter regions of various genes. Although many CcpA-related genes were identified, a relationship between CcpA and *gtfBC*, *spaP*, or *vicRK* was not demonstrated. In addition, we analyzed the expression of *glmS* and *nagB* in the *ccpA* mutant and found that *glmS* expression was decreased upon the addition of GlcNAc, while *nagB* expression was increased. These results suggest that CcpA is not involved in the expression of *glmS* or *nagB* under the specific growth conditions tested.

In conclusion, we demonstrated that the expression of NagB and GlmS in *S. mutans* is tightly regulated and modulated by the presence or absence of GlcNAc in the environment. The failure of NagB and GlmS regulation affected virulence-associated factors such as biofilm formation and saliva-induced aggregation, indicating that sugar metabolism is related to the virulence of *S. mutans*. These observations help explain how *S. mutans* is able to colonize and form dental caries in the oral cavity

## Supporting Information

Figure S1 **NagB and GlmS expression in the knockout mutants and their complementation strains.** After washing WT and mutant cells grown overnight in TSB with or without 10 mM GlcNAc, a small aliquot of each was inoculated into CDM-G50 with or without 10 mM GlcNAc and incubated at 37°C with 5% CO_2_. When the sample reached an OD_660_ of 0.5, the cells were collected. Samples were prepared for immunoblotting (A) and quantitative PCR (B) as described in the Materials and Methods. *p < 0.05, as determined by Tukey’s HSD; **p < 0.005, as determined by Tukey’s HSD.
(TIF)

Figure S2 **TCS expression in the** ***glmS*** **and** ***nagB*** **mutants.** After washing WT and mutant cells grown overnight in TSB with or without 10 mM GlcNAc, a small aliquot of each was inoculated into CDM-G50 with or without 10 mM GlcNAc and then incubated at 37°C with 5% CO_2_. When the sample reached an OD_660_ of 0.5, the cells were collected. Samples were prepared for quantitative PCR as described in the Materials and Methods. HK and RR represent histidine kinase and response regulator, respectively. *p < 0.05, compared to WT as determined by a *t*-test.
(TIF)

Figure S3 **Expression of GlmS, NagB, and virulence factors in WT and mutant UA159 cells in a biofilm.** Biofilm cells grown in CDM-G50 containing sucrose and/or GlcNAc and planktonic cells in CDM-G50 with or without GlcNAc were collected and prepared for immunoblotting (A) and quantitative PCR (B) as described in the Materials and Methods. Panel (B): 1: wild type, 2: *glmS* mutant, 3: *nagB* mutant. *p < 0.05, compared to WT as determined by a *t*-test; ^+^ *p* < 0.05, compared to WT as determined by a *t*-test.
(TIF)

Figure S4 **Determination of the transcriptional start sites by RACE.** The transcriptional start sites of *glmS* (A) and *nagB* (B) were determined by RACE experiments (Fig. S2). RACE was performed with a 5’-Full RACE Core Set (Takara Bio Inc., Shiga, Japan), according to the manufacturer’s protocol. The primers used are listed in Table S1. The black arrow indicates the primers used in our 5’ RACE analyses. The transcription start point (+1) is marked with a bent arrow. The start sites of *glmS* and *nagB* were 87 and 22 bp upstream of the translational start site, respectively. On the basis of this experiment, the predicted promoter regions of both genes were determined. The putative promoter region (–35 and –10) is underlined.
(TIF)

Table S1 **Primers used in this study.**
(DOC)
