# Supplementary figures and images for: GlmS and NagB Regulate Amino Sugar Metabolism in Opposing Directions and Affect Streptococcus mutans Virulence
Source: PLoS One. 2012 Mar 16;7(3):e33382. doi: 10.1371/journal.pone.0033382 (PMC3306399; doi:10.1371/journal.pone.0033382)

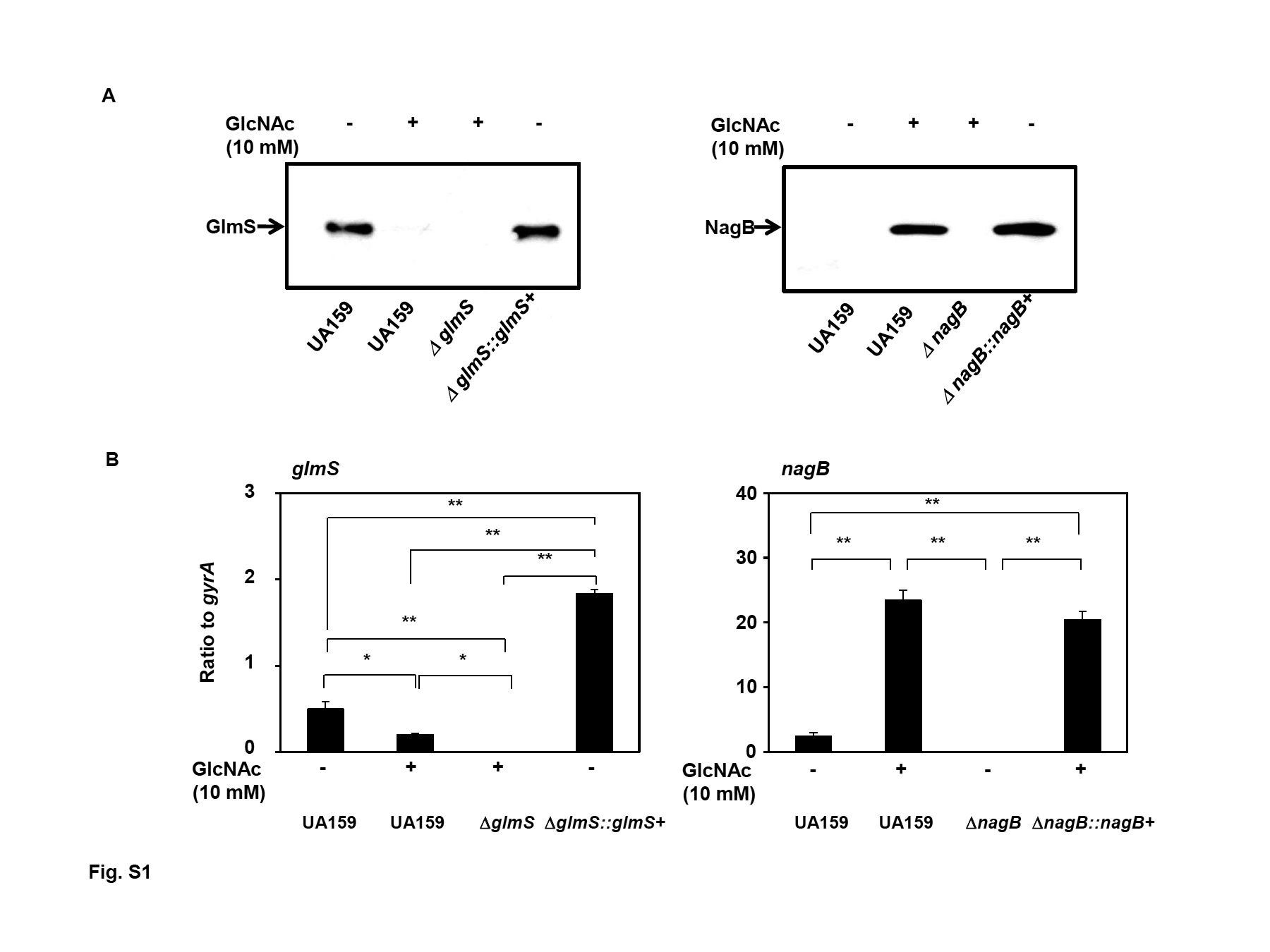

Supplement: Figure S1 — NagB and GlmS expression in the knockout mutants and their complementation strains. After washing WT and mutant cells grown overnight in TSB with or without 10 mM GlcNAc, a small aliquot of each was inoculated into CDM-G50 with or without 10 mM GlcNAc and incubated at 37°C with 5% CO2. When the sample reached an OD660 of 0.5, the cells were collected. Samples were prepared for immunoblotting (A) and quantitative PCR (B) as described in the Materials and Methods. *p < 0.05, as determined by Tukey’s HSD; **p < 0.005, as determined by Tukey’s HSD. (TIF) [file pone.0033382.s001.tif]

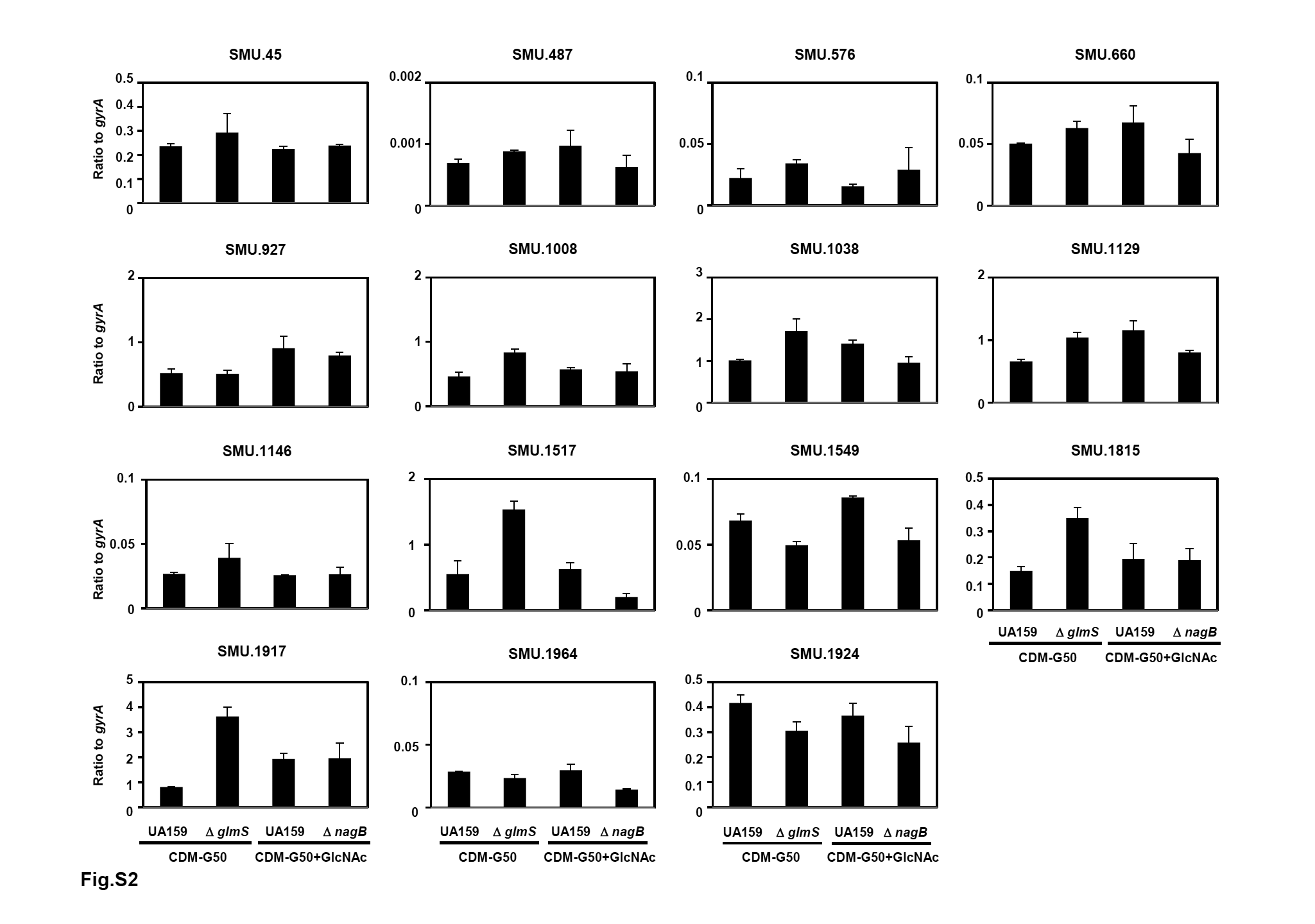

Supplement: Figure S2 — TCS expression in the glmS and nagB mutants. After washing WT and mutant cells grown overnight in TSB with or without 10 mM GlcNAc, a small aliquot of each was inoculated into CDM-G50 with or without 10 mM GlcNAc and then incubated at 37°C with 5% CO2. When the sample reached an OD660 of 0.5, the cells were collected. Samples were prepared for quantitative PCR as described in the Materials and Methods. HK and RR represent histidine kinase and response regulator, respectively. *p < 0.05, compared to WT as determined by a t-test. (TIF) [file pone.0033382.s002.tif]

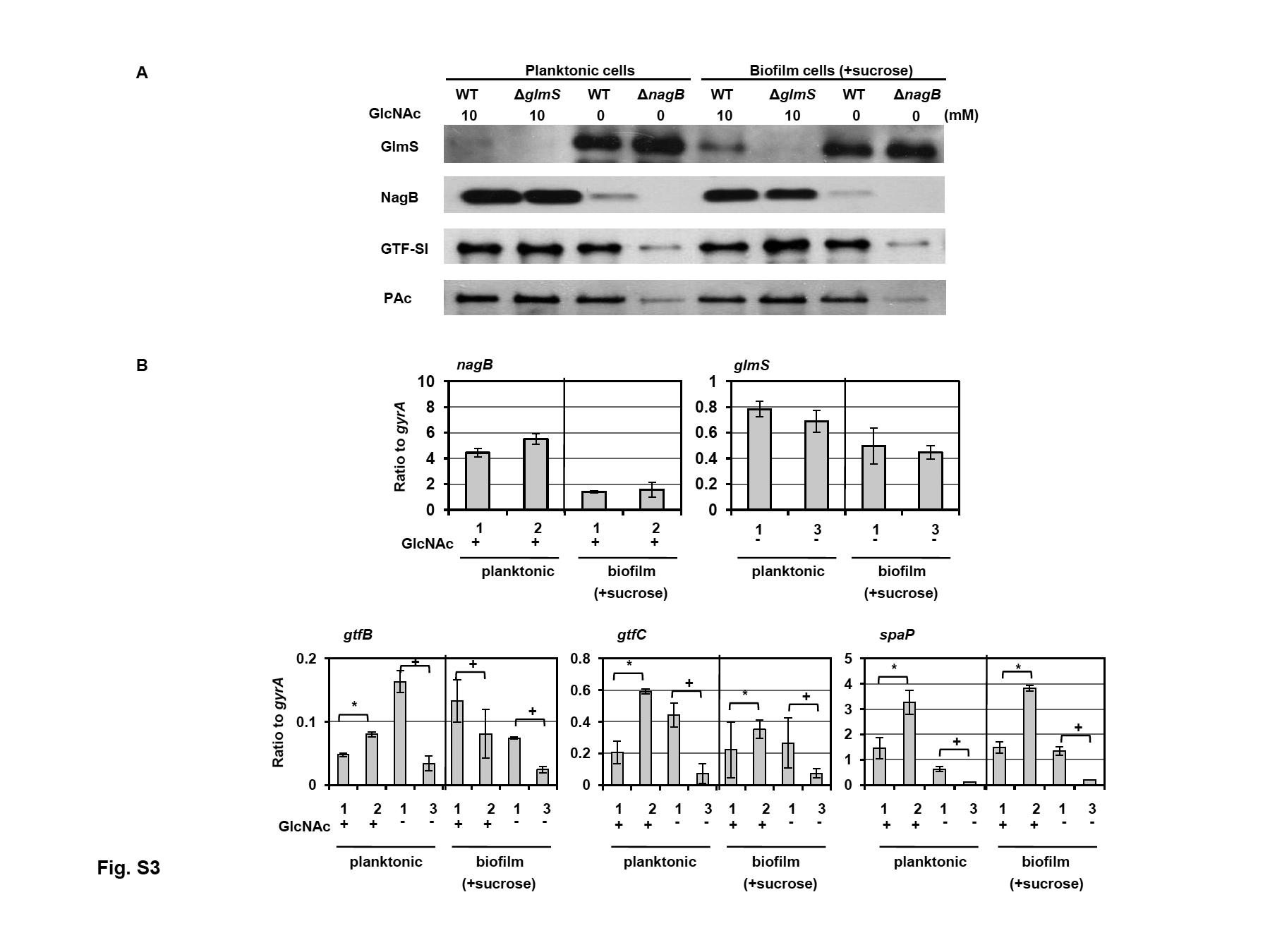

Supplement: Figure S3 — Expression of GlmS, NagB, and virulence factors in WT and mutant UA159 cells in a biofilm. Biofilm cells grown in CDM-G50 containing sucrose and/or GlcNAc and planktonic cells in CDM-G50 with or without GlcNAc were collected and prepared for immunoblotting (A) and quantitative PCR (B) as described in the Materials and Methods. Panel (B): 1: wild type, 2: glmS mutant, 3: nagB mutant. *p < 0.05, compared to WT as determined by a t-test; + p < 0.05, compared to WT as determined by a t-test. (TIF) [file pone.0033382.s003.tif]

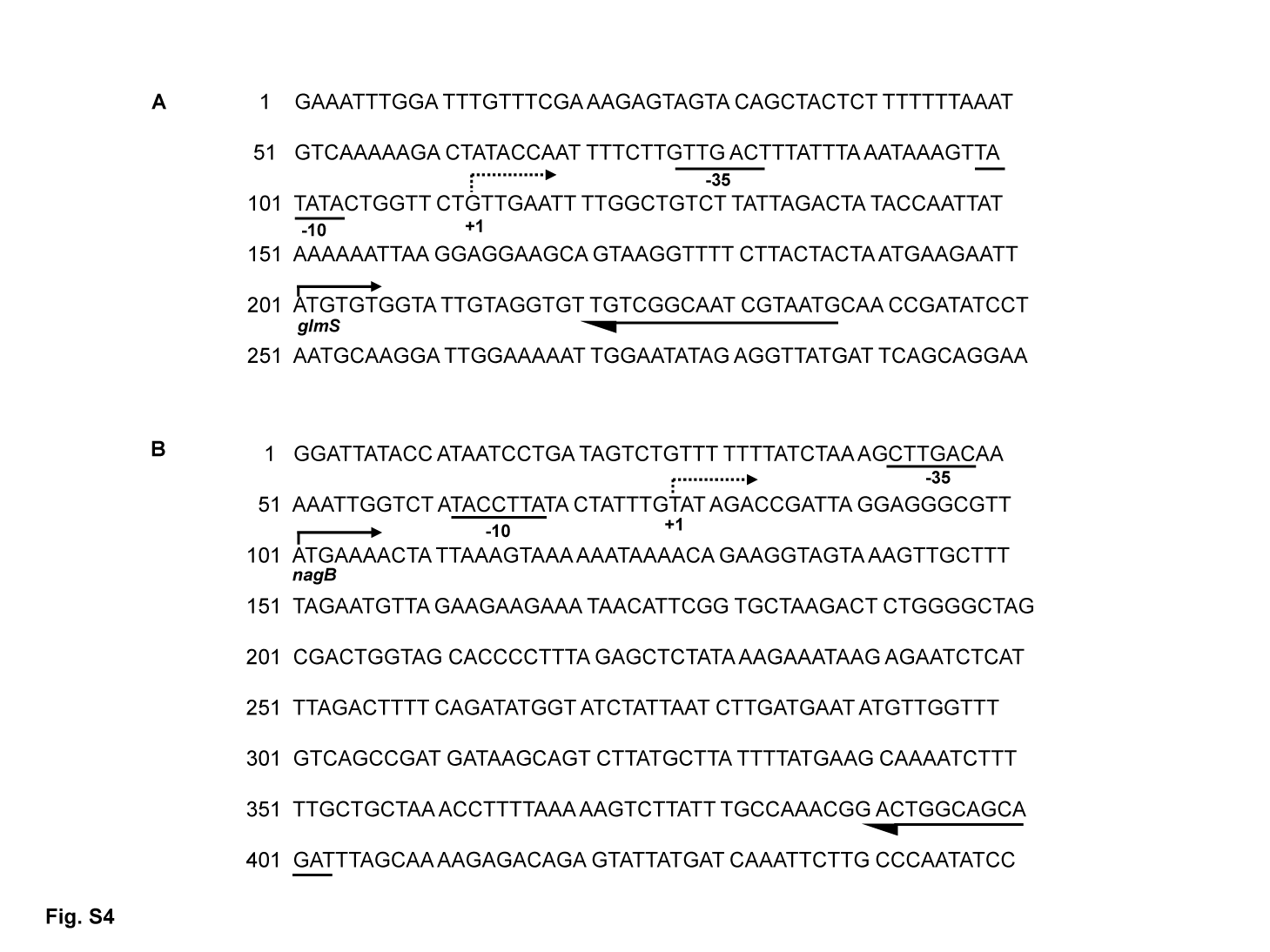

Supplement: Figure S4 — Determination of the transcriptional start sites by RACE. The transcriptional start sites of glmS (A) and nagB (B) were determined by RACE experiments (Fig. S2). RACE was performed with a 5’-Full RACE Core Set (Takara Bio Inc., Shiga, Japan), according to the manufacturer’s protocol. The primers used are listed in Table S1. The black arrow indicates the primers used in our 5’ RACE analyses. The transcription start point (+1) is marked with a bent arrow. The start sites of glmS and nagB were 87 and 22 bp upstream of the translational start site, respectively. On the basis of this experiment, the predicted promoter regions of both genes were determined. The putative promoter region (–35 and –10) is underlined. (TIF) [file pone.0033382.s004.tif]
