# Supplementary material for: GlmS and NagB Regulate Amino Sugar Metabolism in Opposing Directions and Affect Streptococcus mutans Virulence
Source: PLoS One. 2012 Mar 16;7(3):e33382. doi: 10.1371/journal.pone.0033382 (PMC3306399; doi:10.1371/journal.pone.0033382)
Supplement: Table S1 — Primers used in this study. (DOC) [file pone.0033382.s005.doc]

**Supporting Information**

Table S1. Primers used in this study

|  |  | Specific primers for PCR | | |
| --- | --- | --- | --- | --- |
| Gene ID | Gene name | Forward |  | Reverse |
| Quantitative PCR | |  |  |  |
| SMU.1114^1^ | *gyrA* | 5’-tctcgctggacttgtcactg-3’ |  | 5’-catctaggcgcatcactttg-3’ |
| SMU.1187 | *glmS* | 5’-cgaggtgccaatgtctta-3’ |  | 5’-acgttgaagtgaggcatag-3’ |
| SMU.0636 | *nagB* | 5’-atttctatgggactggcctcta-3’ |  | 5’-accttaggatgtgtttgcagta-3’ |
| SMU.1004 | *gtfB* | 5’-accgaagtgacatctaagca-3’ |  | 5’-tccccaacagtataaggatt-3’ |
| SMU.1005 | *gtfC* | 5’-ggcggttacagaatctcagg-3’ |  | 5’-tccgaagttgttgttggttt-3’ |
| SMU.0610 | *spaP* | 5’-ccctgccatctggttatc-3’- |  | 5’-agaacttgtccgaccactg-3’ |
| SMU.0045 | *-* | 5’-atcgagatccagaatatgaagc-3’ |  | 5’-cttgagttgccatagttcgtag-3’ |
| SMU.0486 | *liaS* | 5’-ttaaacatgccaaggctagtc-3’ |  | 5’-gtaactcaaatctcgaacgaca-3’ |
| SMU.0577 | *-* | 5’-tctaaggcaatttgtggct -3’ |  | 5’-tctgactgatcgaaccaatg-3’ |
| SMU.0659 | *-* | 5’-tatgatgcctgatgtggacg-3’ |  | 5’-ccgtgacaacatctgctt-3’ |
| SMU.0927 | *relR* | 5’-ggaagctttgcgtgagat-3’ |  | 5’-agcacctgcctctaaacca-3’ |
| SMU.1008 | *bceR* | 5’-gctatgaatatgggaggtga-3’ |  | 5’-gccttgcttagtgaactcgt-3’ |
| SMU.1038 | *-* | 5’-aactgtcacccgtgcataa-3’ |  | 5’-ttcgcaaagttagctctgttc-3’ |
| SMU.1129 | *ciaR* | 5’-gctgtatcaacggttaactcac-3’ |  | 5’-aggacacggttttgaattagg-3’ |
| SMU.1146 | *-* | 5’-gattcgcaactccaattcct-3’ |  | 5’-ttatgctgcctgatggttc-3’ |
| SMU.1516 | *vicK* | 5’-ctaggagccactgattctgt-3’ |  | 5’-cgccgtctctttgtttcta-3’ |
| SMU.1517 | *vicR* | 5’-aacatcaacagtacggacatc-3’ |  | 5’-gctaaaaaacgtggaacagag-3’ |
| SMU.1548 | *‐* | 5’-ctcttctagttgcttttgcacg-3’ |  | 5’-aaatgagcgcaatcggat-3’ |
| SMU.1815 | *-* | 5’-tgttggggtcagtgatagatgt-3’ |  | 5’-aaggacttgattcaggaggaga-3’ |
| SMU.1917 | *comE* | 5’-caataaaagagcgatggcact-3’ |  | 5’-cccttttgctgagattctgt-3’ |
| SMU.1924 | *gcrR* | 5’-ttgacgacgaaatatggcac-3’ |  | 5’-agagatggacgggtatgaagt-3’ |
| SMU.1965 | *‐* | 5’-aactgcgtcaattcctgctg-3’ |  | 5’-ttggctgagaaaatcgctga-3’ |
| Construction for the mutant | | |  |  |
|  | *glmS*-UP^2^ | 5’-cgcggtaccgtaggtgttgtcggcaat-3’ |  | 5’-cgcctcgagccttcacctagaccgatt-3’ |
|  | *glmS*-DW^3^ | 5’-agggattgcatcggaatg-3’ |  | 5’-cgcccgcggtctcgctccgaaagacta-3’ |
|  | *nagB*-UP | 5’-cgcggtacccttccgctgaacttttaac-3’ |  | 5’-cgcctcgagccctcctaatcggtctat-3’ |
|  | *nagB*-DW | 5’-ggactggcagcagattta-3’ |  | 5’-cgcccgcggtctttgctgcgctggata-3’ |
|  | *ccpA-UP* | 5’-ttccaaggcaattttaga-3’ |  | 5’-cagtcgaggattacgcttccattggtatt-3’ |
|  | *ccpA-DW* | 5’-gctgacctagttcagagaaagaggaacaact-3’ |  | 5’-aatctttctttggcatca-3’ |
| Construction for *glmS* complementation strain | | |  |  |
|  | *ftf*-UP | 5’-tttactaagttcaacaatgg-3’ |  | 5’-aagtcgacccaccaataacattccaat-3’ |
|  | *glmS* | 5’-ggaggatccagtaaggttttcttactact-3’ |  | 5’-tctttgtttcttaaaacagcccaaaaaaacc-3’ |
|  | *ftf*-DW | 5’-aagaaacaaagaaagctcatcatgtttcaac-3’ |  | 5’-cggccgcggttcgtcttgtttctctca-3’ |
|  | *spc* | acaggctcttcgttcgtgaatacatg |  | tgatctaagaatgaatatttcccaaa |
| Construction for *nagB* complementation strain | | |  |  |
|  | *ftf*-N | aatgatatcgtgtttgtt |  | cgaacgaagagcctgttctgttagac |
|  | *ftf*-C | aaaagggtgatgggactacaaaagtc |  | aaccaatgcttacacaga |
|  | *nagB* | attcattcttagatcagacagtcggg |  | gtcccatcacccttttcttccatcag |
|  | *spc* | acaggctcttcgttcgtgaatacatg |  | tgatctaagaatgaatatttcccaaa |
| Construction for recombinant protein | | |  |  |
| SMU.1187 | *glmS* | 5’-cgcggatccatgtgtggtattgtaggtg-3’ |  | 5’-cgcagatctttcaacagtaacagctttag-3’ |
| SMU.0636 | *nagB* | 5’-cgcggatccatgaaaactattaaagtaaaaa-3’ |  | 5’-cgcagatctaattcctgctcctgctt-3’ |
| RACE assay | | |  |  |
| SMU.1187 | *glmS* |  |  | 5’-gcattacgattgccgaca-3’ |
| SMU.0636 | *nagB* |  |  | 5’-atctgctgccagtc-3’ |

1. GenBank locus tag was from the *S. mutans* genome at the Oral Pathogen Sequence Database site

2. UP: Upstream region of *glmS* or *nagB* gene

3. DW: Downstream region of *glmS* or *nagB* gene
